# Estimating uncertainty and reliability of social network data using Bayesian inference

**DOI:** 10.1098/rsos.150367

**Published:** 2015-09-16

**Authors:** Damien R. Farine, Ariana Strandburg-Peshkin

**Affiliations:** 1 Edward Grey Institute of Field Ornithology, Department of Zoology, University of Oxford, Oxford, UK; 2 Department of Anthropology, University of California Davis, Davis, CA, USA; 3 Smithsonian Tropical Research Institute, Ancon, Panama; 4 Department of Ecology and Evolutionary Biology, Princeton University, Princeton, NJ, USA

**Keywords:** social network analysis, uncertainty, Bayesian inference, social structure, interactions, group-living

## Abstract

Social network analysis provides a useful lens through which to view the structure of animal societies, and as a result its use is increasingly widespread. One challenge that many studies of animal social networks face is dealing with limited sample sizes, which introduces the potential for a high level of uncertainty in estimating the rates of association or interaction between individuals. We present a method based on Bayesian inference to incorporate uncertainty into network analyses. We test the reliability of this method at capturing both local and global properties of simulated networks, and compare it to a recently suggested method based on bootstrapping. Our results suggest that Bayesian inference can provide useful information about the underlying certainty in an observed network. When networks are well sampled, observed networks approach the real underlying social structure. However, when sampling is sparse, Bayesian inferred networks can provide realistic uncertainty estimates around edge weights. We also suggest a potential method for estimating the reliability of an observed network given the amount of sampling performed. This paper highlights how relatively simple procedures can be used to estimate uncertainty and reliability in studies using animal social network analysis.

## 1. Introduction

Social networks are widely used to investigate the social structure of animal populations [1–5]. Quantifying the patterns of associations or interactions among individuals can yield novel insights into their evolutionary consequences [6,7]. However, a challenge in applying network methods to animal systems is that we often have to contend with small and incomplete sampling [5,8]. Small sample sizes lead to uncertainty in the existence and strength of associations or interactions, and at present there are limited tools available for incorporating this uncertainty into network analyses [9,10]. Further, there are no existing methods for estimating whether a set of observations reliably captures the structure of the real set of interactions (or the real network). As a result, most studies are limited to assuming that their observations are representative of the underlying social structure, and rarely evaluate the potential error in their network estimates. In this paper, we develop an approach based on Bayesian inference to estimate rates of association or interaction between pairs of individuals, as well as their uncertainty. We compare our method to an existing approach of computing index-based social networks from observed data and estimating uncertainty using bootstrapping [9–11]. We then demonstrate how both these methods can be used to determine how accurately the associations among individuals and social network properties have been captured in empirical studies.

A network is defined as a set of *nodes* that are connected by a set of *edges*. In animal social networks, nodes typically represent individual animals, and edges represent either interactions (such as grooming) or associations (based on proximity or co-occurrence in space) between them [5,10,12]. In many cases, edges are weighted, with the *edge weight* representing the *interaction rate* or the *association rate* between a pair of nodes (known as a *dyad*). Social network data often comes in the form of a set of *observations* of dyads, where each *observation* is a record of whether the dyad was observed interacting/associating or not. We can therefore represent observations of each dyad as a set of 1s and 0s, where 1 indicates that the individuals were observed to be interacting or associating, and 0 indicates that they were observed to be not interacting or were not seen together. From these binary data, we then would like to estimate the *edge weight* (representing either the *association rate* or the *interaction rate*) for each dyad, which typically captures the rate at which the individuals associate or interact over time [13] and is often calculated using indices (for example by using the simple ratio index (SRI) [14]). In our case, we would also like to estimate the *uncertainty* of these edge weight estimates.

Bootstrapping is a widely used approach for dealing with uncertainty in ecological data [15]. In the non-parametric bootstrap, the distribution of a statistic is estimated from its distribution in pseudosamples obtained by resampling with replacement from the original data. In other words, the observed data are resampled to create new datasets that match the size of the original data, while allowing the same observations to be drawn multiple times. This creates slightly different datasets each time, but always based on the same original observations. Repeating this process hundreds of times and re-calculating a given statistic for each new dataset generates a distribution of possible values. Lusseau *et al*. [9] suggested that this approach could be incorporated into social network analysis. In the case of networks, the observation data from which the observed network was generated is bootstrapped (observations are resampled, rather than resampling nodes) and a new network is generated for each dataset by re-calculating all the edge weights in exactly the same way. The statistic of interest in the observed network is re-calculated each time and recorded. The 95% confidence interval can then be inferred by extracting the 2.5% and 97.5% quantiles of the recorded values. However, an issue with this approach is that it relies on having good observation data in the first place. Bootstrapping methods may underestimate uncertainty and lead to biased estimates when sample sizes are very small, as is often the case in animal social network data. For example, in the limiting case where there is only one observation, a bootstrapping method would draw this value in every sample and would conclude that the uncertainty around this estimate is zero.

An alternative approach to bootstrapping may be to estimate edge weights using Bayesian inference. In a Bayesian statistical approach, a distribution of values is generated for each variable. In this case, we suggest that a distribution (known as the posterior distribution) can be generated for each edge weight (i.e. association rate) in the network. This distribution captures both the most likely value of the edge weight and the uncertainty around that value. From this distribution, one can extract the 95% credible interval (the Bayesian equivalent of the confidence interval, defined as a region containing 95% of the probability). From the collection of posterior distributions for all edges, it is also possible to derive a number of candidate networks and estimate the uncertainty associated with a given network statistic across these. Previous work has applied Bayesian inference to human social networks in order to estimate the existence or absence of network edges when there are incorrect observations [16]. However, that method was designed to estimate the presence or absence of binary edges, rather than the posterior probability density of edge weights (typically representing association or interaction rates between individuals) that is missing from studies on animal social networks.

A related question in the analysis of animal social networks is whether it is possible to determine if a set of observations, and the resulting inferred network, are sufficient to reliably estimate properties of the real social structure. Answering this question is critical, particularly when sample size is limited. If network estimates are unreliable, this could lead us to draw incorrect conclusions about the structure of animal societies. Yet, to date, there are no established methods for estimating if a network is a good representation of the real life social structure. A Bayesian approach may be particularly useful in addressing this challenge. Edge weights calculated using indices [14], which are commonly used in animal social network analysis, perform poorly at quantifying uncertainty when data are sparse (few observations are available) because many edge values will be binary. That is, for a pair of nodes that has only been observed once, the index-based edge weight will be either 0 or 1 and will not have any uncertainty under a bootstrapped estimate. The aim when constructing networks is to refine the estimate of edge weight values by using many observations of the same pair of nodes together or apart (e.g. after two observations, edge weights can only have values of 0, 0.5 or 1, whereas edges observed 20 times can have values in the set {0,0.05,…,0.95,1}). The aim of quantifying uncertainty in edge weight estimates should be to provide a realistic estimate that reflects the sampling effort (and one that is valid given any number of samples). Estimating edge weights using a Bayesian approach uses an initial distribution of possible values for each edge (i.e. the prior distribution) that is refined after every observation. This approach commences with a large credible interval when few observations are made. When more observations become available, this should result in a decrease in the credible interval for a given edge weight.

In this paper, we present a Bayesian-based approach for estimating the potential distribution of edge weights for each dyad in a network given a set of observations. This complements a growing body of work developing models to extract networks from noisy or incomplete data [17,18]. We explore how this method performs at estimating edge weights in the real social network under different amounts of sampling, compared to the standard method of using simple probabilities of dyadic co-occurrences. We also evaluate the accuracy of uncertainty estimates generated from this Bayesian-based procedure, as compared to the accuracy of uncertainty estimates generated by bootstrapping the observed data as recently suggested by Lusseau *et al*. [9]. We then extend our method to estimate the values and uncertainty of higher-order network properties, and again evaluate its performance compared to the bootstrapping method. Finally, we explore a potential method based on subsampling for determining whether an observed network is a robust estimate of social structure.

## 2. Material and methods

### 2.1 General procedure for estimating uncertainty in edge weights

To investigate how uncertainty can be incorporated into network analyses, we ran the following steps:

(1) Generate a ‘real’ network.
(2) Sample from this network *S* times to generate binary ‘sampling periods’, i.e. sets of observations, where each dyad is observed to be either associating (1) or not associating (0).
(3) Aggregate the data from these sampling periods to estimate edge weights and uncertainties for each dyad (using one of two methods—see below).

We repeated this process for 100 instances of four types of networks. For the four classes, we varied the number of individuals (15 or 50 nodes) and whether the network contained local structure (either with cliques of strongly inter-connected individuals or with no cliques, see below).

#### 2.1.1 Generating the ‘real’ networks

We developed a simple algorithm for generating a ‘real’ network. The network consisted of either 15 or 50 individuals. To capture the fact that individuals often vary in their sociality, each individual was allocated a gregariousness score that was calculated by drawing a value from a Poisson distribution (λ=3) and adding 0.05 (to avoid creating isolates). This models the fact that degree is generally Poisson distributed in animal social networks. We then turned this gregariousness score into a probability of being gregarious by dividing all individuals' scores by the largest value. For networks with no cliques, in which individuals associate equally with all others, we set the probability of a given dyad associating in a given time period to 0.5. For networks with cliques, individuals were allocated to one of five different communities with equal probability, with a within-clique association probability of 0.9 and a between-clique association probability of 0.1. We then calculated a weight for each edge, which we defined as the product of the two individual's gregariousness scores, multiplied by the probability defined by being in the same or different cliques (if present). Thus, two individuals that have a high gregariousness score and occur in the same clique get a large edge value, whereas two individuals that have low gregariousness scores and are from different cliques get a weak edge value, with variation among individuals shaped by a Poisson process.

#### 2.1.2 Generating ‘observation’ samples

For a given sampling effort, *S*, we generated *S* sampling periods. Each sampling period consisted of an ‘observed’ network in which pairs of individuals were detected either as having been observed together or not [10,19]. The values of edges (either 0 or 1) for each observed network were drawn at random, with the probability of drawing a 1 (the two individuals are observed together during that sample) equal to the edge weight in the real network. Thus, dyads with large edge weights (those that associate frequently) more often have 1s across sampling periods.

#### 2.1.3 Estimating edge weights and their uncertainty using the simple ratio index and bootstrapping (b-SRI method)

We used the *asnipe* [20] package in R [21] to create a network (N×N matrix) from each set of observations (collection of sampling periods). The weights of edges in these networks represent the probability that individuals were observed together given that at least one was observed during the sampling period, which is also known as the SRI [14]. This is a widespread and common way to estimate interaction or association rates [5,10], and in this scenario produces edge weights that capture the association rate.

Using the same sampled data, we then estimated the uncertainty in our edge weights using a bootstrapping procedure. For each set of observations, we created 100 bootstrapped networks by randomly selecting *S* sampling periods from the observed data with replacement, thus creating new, but slightly different, sampling period data each time. For each bootstrap replicate, we computed the association rate (SRI) of all dyads, creating a new network that differed slightly from the observed network. This procedure allowed us to estimate the uncertainty of the edge weight for each dyad, and also to compute the uncertainty of network-level properties by calculating network metrics for each of the bootstrapped networks.

#### 2.1.4 Estimating edge weights and their uncertainty using Bayesian inference

**Figure 1.**
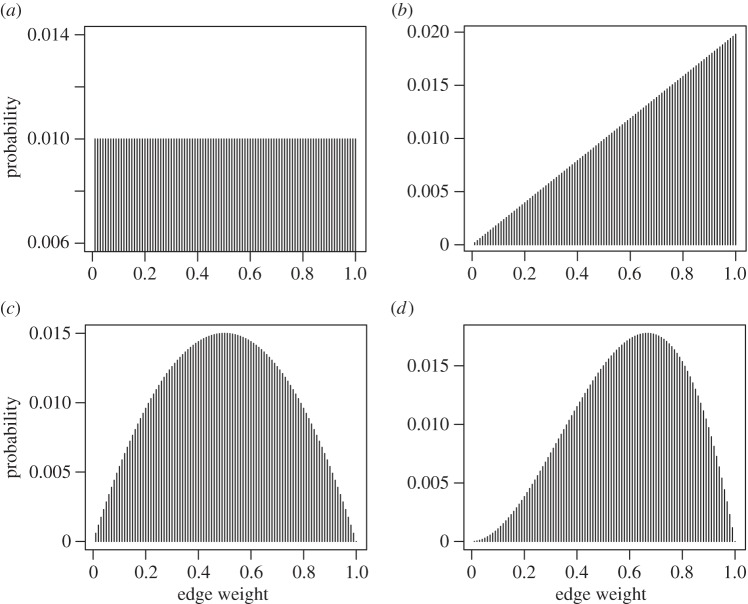
Toy example of Bayesian updating for a single edge in a network. After each observation, the estimated probability distribution of a given edge weight is updated based on whether the individuals were associated or not. Starting with a uniform prior (*a*), the distribution is updated when the individuals were associated in the first observation (*b*), then absent in the second (*c*) and finally present in the third (*d*).

Based on the data from our sampling periods, we also estimated dyadic edge weights and their uncertainty using Bayesian inference. For each edge, we began with a prior distribution of edge weights (see below for information on how we constructed this prior). We then updated this distribution using Bayesian inference. This method combines prior estimates of associations/interactions with observation data to produce a probability distribution of the value for each edge. A toy example, starting with a uniform prior, is shown in figure 1.

To avoid issues of binning and to improve computation speed, we used beta conjugate updating given by:

$$P(\theta |d,s)=\theta^{d+a-1}{\left(1-\theta \right)}^{\left(s-d\right)+b-1},$$

where *θ* is a given edge weight, *d* is number of co-occurrences (1s in the sampling period data), *a* and *b* are the prior values of the parameters in the beta distribution, and *s* is the number of times the dyad was sampled (i.e. the number of times at least one individual was observed). This method assumes that the posterior distribution of edge weights is beta distributed and effectively fits the parameters of this distribution for each dyad based on the observed data.

An important consideration in all Bayesian-based methods is the choice of a prior. In the case of social networks, this prior should reflect the distribution of edge weights that we expected to observe before the data were collected. The best way to arrive at this prior would be by estimating the distribution of edge weights from a past study on the same or a similar system. However, in practice having this type of previous data is uncommon. A second option is to estimate a prior by computing the edge weight distribution from a subset of the observed data, and then leaving this subset of data out when estimating the final network edges. However, due to the small sample sizes that often characterize studies of animal social networks, this method may again be impractical. For the current analysis, we chose to employ an empirical Bayesian method that estimates the parameters of the prior beta distribution from all observed data using maximum-likelihood estimation (type II maximum likelihood). Here, to construct the prior, we maximize the probability of all observations as a function of the parameters of the prior beta distribution, marginalizing the unknown edge weights. While not strictly Bayesian (because the prior incorporates the observed data), this method is widely used in practical applications, and can be viewed as an approximation to a hierarchical Bayesian approach [22], pp. 171–173. Details are provided in the electronic supplementary material.

After specifying the prior (in this case the parameters of a beta distribution), we then use Bayesian inference to compute the posterior distribution of the edge weight for each dyad given a set of observations (1s and 0s across all the sampling periods). From the posterior, we estimate the edge weight between a pair of individuals (the edge weight with the highest probability, or the peak of the distribution) and the uncertainty of this estimate. The posterior distribution of each edge can be used to estimate the 95% credible interval of the edge weight for each pair of individuals.

### 2.2 Evaluation of inferred edge weights

How well did our estimated edge weights capture the true edge weights (in our case association rates) of the networks from which we drew our observations, and how accurately did we capture their uncertainties? We evaluated the performance of the two methods as a function of the increasing number of samples using the following metrics:

(1) The absolute error: the absolute difference between inferred edge weights and their values in the real network.
(2) The relative accuracy: how well-estimated edge weights correlated with their values in the real network (rank correlations). Relative accuracy is important in studies of animal social networks because we often care more about the relative rankings of association strengths or other social traits within a given network than we do about their absolute values.
(3) The reliability of uncertainty estimates: the rate of false positives (where the real value lies below the estimated 95% interval) and false negatives (where the real value lies above the estimated 95% interval). If the calculated 95% intervals are reliable, false positive and false negatives should both be approximately 2.5%.

#### 2.2.1 Determining the absolute error of edge weights in estimated networks

We first estimated the absolute error of edge weights in the inferred networks as a function of the number of samples *S* (i.e. observations). We defined the *error* of each edge weight as the absolute difference between the edge value in the ‘real’ network and the estimated edge value based on the standard SRI metric or Bayesian inference. We then computed the *mean error* across all edge weights within the network. We repeated this procedure across all 100 example networks to obtain 100 mean error values for each value of *S* (the number of observations/samples) for each method. From these, we extracted the mean and the 95% range. Low values of the error indicate that a given method is accurately capturing the values of edge weights in the true network.

#### 2.2.2 Determining the relative accuracy of edge weights in estimated networks

We measured the relative accuracy of edge weights by computing the rank correlation between inferred and real edge weights within a given network. This value should approach 1 if the relative ranking of edge weights is accurately captured in the inferred networks. We measured the relative accuracy for all 100 replicate networks of each type, at each sampling effort *S*, and computed the mean and 95% confidence interval for each value of *S*.

#### 2.2.3 Determining the false positive and false negative rates of estimated networks

While accurately capturing the actual value of edge weights is crucial, our primary aim here is to reliably estimate the uncertainty range for each edge value. To determine how accurately the uncertainty had been estimated for each method, we calculated the number of false positives and false negatives in each estimated network. False positives were defined as edge weights whose real value fell below the 95% interval that was estimated for that edge. Similarly, false negatives were defined as edge weights whose real value fell above the 95% interval estimated for that edge. A large number of false positives or negatives would indicate that the 95% intervals are inaccurate and are not useful for estimating uncertainty in our data. By contrast, values that are too small suggest that the 95% interval is too large or conservative (i.e. we are overestimating the uncertainty in our edge weight measurements). If the 95% intervals are reliable, we expect a false positive and false negative rate of approximately 2.5%. We calculated the number of both false positives and false negatives for each method (the b-SRI and Bayesian methods), as a function of the number of samples *S*. We also compared these values to a third method for inferring 95% intervals (the Clopper–Pearson method [23]), which is expected to provide better estimates of confidence intervals than bootstrapping procedures when data are sparse.

### 2.3 Estimating the values and uncertainty of node-level network metrics

Ultimately, we want to estimate not only edge weights and their uncertainty, but also the values and uncertainty around higher-order metrics quantifying the position of nodes in a network. We therefore extend our Bayesian method to estimate uncertainty of node-level properties and compare this to the results of attaining confidence intervals through bootstrapping. For each network, we computed three node-level network properties: weighted degree, weighted eigenvector centrality and weighted betweenness. Weighted degree is defined for each node in a network as the sum of all of its edge weights, which generally represents how social, or gregarious, it is. Weighted eigenvector centrality represents a generalization of degree that is often used to measure how ‘important’ a node is within a network. High eigenvector centrality usually corresponds to having a high degree and being connected to others with a high degree. Weighted betweenness relates to how many shortest paths cross through a given node and represents relative importance in connecting otherwise disparate parts of a network together (by shortening the path length between other nodes). The use of these metrics in animal social network analysis is discussed further in Farine & Whitehead [5].

To estimate uncertainty on node-level properties, we generated 100 replicate networks for each method either by bootstrapping the sampled data (for the b-SRI method) or by drawing edge weights from the posterior edge weight distribution (for the Bayesian method). We computed the relevant metric for each node within the network and repeated this process 100 times to generate a distribution of values. We then computed the 95% interval for these distributions to estimate uncertainty ranges for each method, at each sampling effort *S*. Finally, we evaluated the absolute and relative accuracy, and the reliability of uncertainty estimates in the same manner as above.

### 2.4 Estimating the reliability of empirical data

In the above analyses, we compared our estimates of node-level and edge-level network properties to the real values of these properties. However, in practice, it is clearly not possible to observe the ‘real’ underlying network from which the observations are drawn. Is there some way to determine whether we have sampled enough that our estimated networks approach the real one? One way to determine the reliability of a network statistic is to observe when our estimate stabilizes as we increase the number of observations. For increasing levels of sampling effort *S*, *S* samples are drawn from the observed data, and the value of the network property (e.g. mean degree) is calculated using one of the two methods described in this paper (b-SRI or Bayesian inference). The distribution (median and 95% range of the posterior) at each level of subsampling can then be used to determine whether the network property is stabilizing by the time it reaches the actual sampling effort. If so, this suggests that estimating network properties from the observed data will generate reliable information about the underlying network. If not, better go collect more data!

To test this idea, we calculated the value and 95% intervals of the edge weights as a function of the number of samples using an empirical dataset containing 347 observations of birds forming flocks. These data are freely available in the R package *asnipe* [20]. Birds in Wytham Woods, UK, were fitted with passive integrated transponder tags, each containing a unique code that was recorded when birds landed on a feeder fitted with radio frequency identification data loggers. The data consist of 151 individuals of five passerine species: 78 blue tits (*Cyanistes caeruleus*), seven coal tits (*Periparus ater*), 51 great tits (*Parus major*), 11 marsh tits (*Poecile palustris*), three nuthatches (*Sitta europaea*) and one individual of unknown species. Data were collected from four feeders, spaced approximately 300 m apart, over the course of one day. Detections were assigned to foraging flocks using an established method based on Gaussian Mixture Models [24,25] that extracts temporally variable bursts of visits by birds (see also [1,26–28]). In the current study, we calculated the edge values (using the SRI) and uncertainty (using the bootstrap, Clopper–Pearson and Bayesian methods) for increasing numbers of observed groups (in increments of 20 observed flocks). We also calculated the mean weighted degree across all individuals as a function of sample size.

## 3. Results

We found that both the Bayesian inference method and the bootstrapped SRI (b-SRI) methods produced useful information about the uncertainty in an association network. Incorporating uncertainty at the level of an edge value enabled us to address two key questions: (i) how well do b-SRI and Bayesian network methods perform at capturing the structure of real underlying networks, and (ii) how can we determine from observation data alone whether a network has been sampled enough to provide accurate results?

### 3.1 Accuracy of inferred edge weights

**Figure 2.**
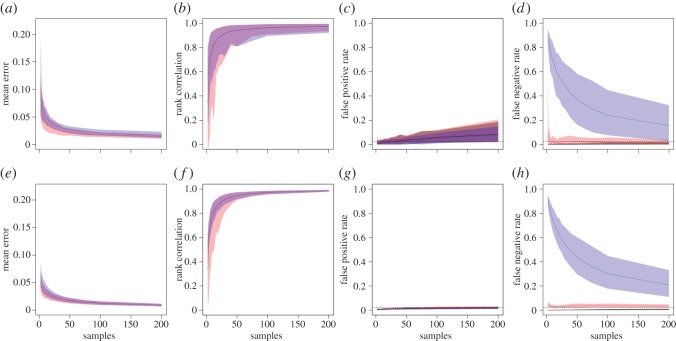
Summary of edge weight accuracy and reliability of uncertainty estimates for small (*N*=15, top row) and large (*N*=50, bottom row) networks with cliques using Bayesian (red) and b-SRI (blue) methods from 100 simulated networks (shown are the median and 95% range). Mean error (*a*,*e*) is the mean absolute difference between the estimated edge weight and the input edge weight in the ‘real’ network for increasing number of samples (*x*-axis). Rank correlation (*b*,*f*) is the correlation in the order of edge weight values, which we call relative accuracy. False positive rate (*c*,*g*) is the proportion of edges in the ‘real’ network that fall below the 95% confidence (bootstrap in blue and Clopper–Pearson in grey) and credible (Bayesian in red) intervals. False negative rate (*d*,*h*) is the proportion of edges in the ‘real’ network that fall above the 95% range for each method for estimating uncertainty. Results for networks with no cliques are shown in the electronic supplementary material, figure S1.

As expected, the accuracy of the edge weights inferred from both the b-SRI and Bayesian inference networks increased with sampling effort (figure 2). This result is consistent with a previous study that investigated the effects of sampling effort when data were captured using gambit of the group rather than sampling periods [29]. As sampling effort increased, the accuracy of the edge weight estimates for both the b-SRI and Bayesian methods increased sharply at first, then tapered off for large sample sizes. In all cases, the increase in accuracy began to taper off after approximately 20 complete samples of the network, suggesting that this might represent a good level of sampling necessary to generate reliable estimates of edge weights (at least in our example networks). However, even for low sampling effort, the mean error of edge weights generally fell below 0.1, indicating that both methods generated fairly reliable estimates even when incorporating only a few observations of each dyad. In general, the Bayesian method tended to perform slightly better than the b-SRI method at accurately capturing the values of edge weights (figure 2*a*,*e*). Both methods were also able to accurately capture the relative rankings of edge weights within the real networks, given sufficient sampling effort (figure 2*b*,*f*).

### 3.2 Reliability of inferred 95% intervals on edge weights

The level of false positives and false negatives (real edge weights that fell outside the 95% interval generated from the observed data) differed strongly between the Bayesian and b-SRI methods (figure 2*c*,*d*,*g*,*h*). In particular, the b-SRI networks resulted in many more false negatives than the Bayesian networks, i.e. they were more likely to significantly underestimate the edge weights for pairs of nodes. This problem was exacerbated for low sampling efforts, because an edge weight is taken to be equal to 0 until the dyad is observed at least once. This illustrates a general issue with bootstrapping methods, which is that they are prone to underestimating uncertainty ranges when sample sizes are small. By contrast, the Bayesian networks, while having slightly higher false positive rates (overestimating the true edge weight), remained largely constant, and close to the expected value of 2.5%, across a range of sampling efforts. Although it may seem detrimental to have a greater rate of false positives, these edges will generally have low edge weights, and weighted network measures can help account for their effect. By contrast, there is no way to account for the absence of an edge when testing a hypothesis using an index-based edge definition (such as the SRI). Overall, the Bayesian method strongly outperformed the b-SRI in terms of accurately estimating the 95% intervals on edge weights, giving reasonable error ranges that resulted in false positive and false negative rates near the expected value of 2.5%.

One non-Bayesian way to circumvent the issue of underestimating error when using small sample sizes is to use an exact small-sample confidence interval on a binomial proportion, such as the Clopper–Pearson interval [23]. In our networks, we found that the Clopper–Pearson interval generated more reasonable confidence interval estimates than the bootstrapping method, in particular by dramatically reducing the false negative rate (figure 2*d*,*h*). However, this method also tended to be slightly too conservative, often overestimating the 95% confidence intervals with false negative rates substantially below the expected 2.5% value.

### 3.3 Accuracy of node-level network metrics and reliability of their 95% intervals

**Figure 3.**
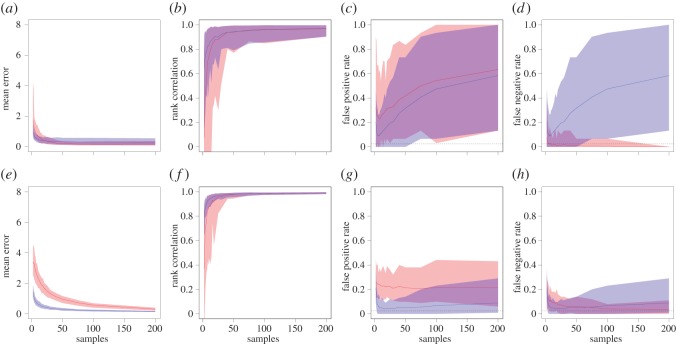
Summary of degree accuracy and reliability of uncertainty estimates for small (*N*=15, top row) and large (*N*=50, bottom row) networks with cliques using Bayesian (red) and b-SRI (blue) methods from 100 simulated networks (shown are the median and 95% range). Mean error (*a*,*e*) is the mean absolute difference between the estimated node degree and the degree in the ‘real’ network for increasing number of samples (*x*-axis). Rank correlation (*b*,*f*) is the correlation in the order of degree scores, which we call relative accuracy. False positive rate (*c*,*g*) is the proportion of node degree values in the ‘real’ network that fall below the 95% confidence (bootstrap in blue and Clopper–Pearson in grey) and credible (Bayesian in red) intervals. False negative rate (*d*,*h*) is the proportion of node degree values in the ‘real’ network that fall above the 95% range for each method for estimating uncertainty. Results for networks with no cliques are shown in the electronic supplementary material, figure S2.

At the node level, the b-SRI and Bayesian network methods both were able to accurately capture both the absolute (figure 3*a*,*e*) and relative (figure 3*b*,*f*) values of weighted node degree after sufficient sampling. Both methods approached a correlation of 1 with the real network's values after approximately 20 complete samples of the network, with the b-SRI method converging slightly faster both in terms of absolute and relative accuracy. However, neither method produced reliable 95% interval estimates (figure 3*c*,*d*,*g*,*h*). The results for weighted eigenvector centrality had similar tendencies (electronic supplementary material, figure S3), with the b-SRI method slightly outperforming the Bayesian method in terms of uncertainty estimates. We found that the Bayesian networks generally performed better than the b-SRI networks at estimating the betweenness scores of nodes in the network (electronic supplementary material, figure S4). However, both yielded fairly low correlations when compared to betweenness measured on the real network. This could suggest that betweenness centrality is an unstable metric, but we suggest that in this case it arose from the fact that our real networks were fully connected and relationships were poorly differentiated. In most animal social networks, the sampled population will contain some individuals that move between groups more often than others (introducing a spatial component), and individuals will vary strongly in their relationships (i.e. there are often many missing edges). Together, these should lead to more reliable estimates of betweenness than in our simulated networks.

The overall level of accuracy in estimating the properties of the real underlying network suggests that both methods are able to reliably capture the true network structure given enough samples. For the networks we investigated, we found that sampling efforts above approximately 20 samples per edge led to reliable and stable results for all metrics (with the exception of betweenness centrality, as discussed above). However, neither method was able to reliably estimate 95% intervals over all network metrics and network types, suggesting that future work should focus on addressing this problem.

### 3.4 Estimating how well a network represents an unknown ‘real’ network

**Figure 4.**
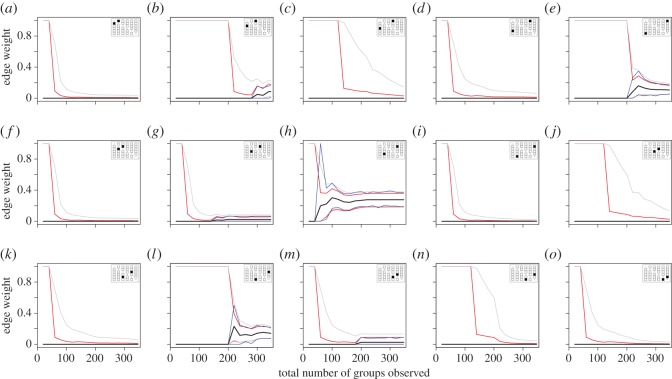
Sample of uncertainty estimates for 15 edges in a mixed-species social network of birds based on the Bayesian method (red), the bootstrap method (blue) and the Clopper–Pearson method (grey). Each plot shows the 95% confidence or credible interval and the edge weight based on the SRI (black line), for increasing number of observed flocks (up to 347). The matrix in the top-right of each plot identifies which edge is being represented in the association matrix (in this case, a symmetrical matrix). For an example using simulated data, see the electronic supplementary material, figure S5.

The Bayesian inference method provided a realistic estimate of uncertainty in individual edge weights (figure 4), starting with an interval ranging from 0 to 1, and rapidly funnelling as observations are made (see electronic supplementary material, figure S5, for the same method applied to simulated data). The Clopper–Pearson confidence intervals showed a similar pattern, but were generally more conservative. By contrast, the b-SRI method had poor estimates of uncertainty when few observations were made (the range for most dyads was 0 to 0). However, visualizing the changing edge weights as the number of samples increases suggests that, for the small sample of 15 dyads shown, most dyads were sufficiently sampled, but that some (e.g. figure 4*b*) may require additional data (the uncertainty around the edge weight is increasing). Notably, the confidence or credible intervals for all three methods converged in most cases, which suggests that, given enough data, the uncertainty for edge weights can be accurately estimated.

**Figure 5.**
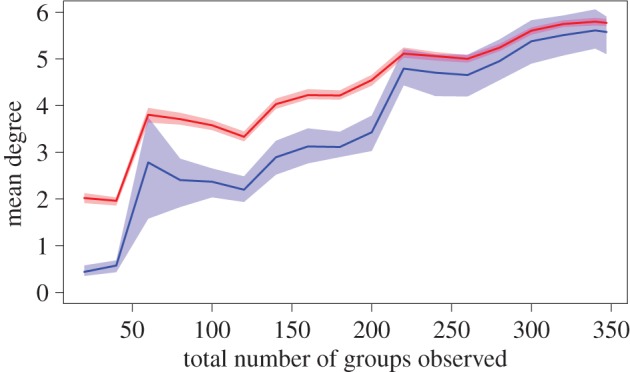
Mean degree in the empirical social network for increasing number of observed groups (using b-SRI method in blue, and Bayesian method in red). The increasing mean degree as more observations are added suggests that too few observations were made. However, the increasing stability (i.e. flattening out) of the metric suggests that not many more observations may be required.

Unlike estimates of edge weights, the mean weighted degree does not stabilize by 347 observations (figure 5), in contrast to what is expected from well-sampled data (electronic supplementary material, figure S6). This suggests that, although in many cases the edge values may be representative of the stable value, in many other cases the edge values still fluctuate as more observations are added. Since mean weighted degree is a property of the whole network, it could be sensitive to under-sampled network edges, especially if under-sampling leads to a bias towards underestimating edge weights.

## 4. Discussion

We propose several new directions for animal social network analysis. First, we demonstrate how Bayesian inference can be used to reliably estimate the uncertainty of each edge in a network. Second, we demonstrate how estimated edge weights and their associated uncertainty ranges can be pooled to estimate the value and uncertainty of higher-order network statistics, although the estimate of uncertainty in these cases may be less reliable. Third, we tackle the issue of estimating robustness and accuracy of an observed network when the true underlying patterns are unknown. We propose subsampling the data to test if progressively increasing the amount of data leads to the stabilization of a given network measurement. If a network is sampled well, then the value of the network statistic should remain stable as more data are added to the analysis.

One limitation of our current method is that it does not account for the possibility of observation errors. When sampling real social networks, errors such as not seeing an individual or mistaking its identity can lead to incorrect observations. In the case of Bayesian inference, one possible drawback is that a single observation of an edge results in setting the probability of that edge not being present to 0. In contrast, if the individuals were frequently observed (not associating), then the bootstrap method will generate many resampled networks where the edge weight is 0. As a result, if there is a non-trivial likelihood of having identification errors in the data, then the Bayesian approach could consistently over-estimate edge weights for the individuals involved. This method also has a tendency to over-estimate degree in larger networks (e.g. *N*=50, figure 3*e*) because of the additive effect of including many edges with very small values that should otherwise be 0. There are a number of potential modifications that could be implemented to address these, and other, issues. Because edge weights in a Bayesian framework represent probability distributions, additional information could be included to help refine the estimates. For example, a hierarchical Bayesian approach could include data on home-range overlap, phenotypic traits, family structure or social clustering when constructing estimates. This could be a powerful approach that would provide not just estimates of association patterns, but would also allow quantification of how phenotypic or state-based drivers underpin population-level social structure (e.g. by quantifying the relative importance of spatial and social drivers [30]). Similarly, the probability of introducing false positives could be modelled and included in the edge weight estimations.

Another source of potentially significant error in social network analysis is observation bias. If different classes of individual consistently vary in how easily observed they are, this can introduce differences in individuals' network properties that do not exist in the real patterns of interaction. One simple example would be if males and females are sexually dimorphic, and the bright males are rarely missed. In such a network, it is highly likely that males will emerge as having a higher degree centrality [5]. This highlights a distinction between estimating uncertainty in social network measures and controlling for potential biases during hypothesis testing. We re-iterate [5,13,31–34] that permutation tests are essential in animal social networks to account for both known and unknown biases during hypothesis testing. However, the Bayesian approach could also be useful for resolving issues with biased edge weight estimations if it is important to accurately estimate the network structure itself (rather than just controlling for it when hypothesis testing). Because the posterior distribution is a probability distribution, then information about the observability of different nodes can be incorporated into the Bayesian model. For example, if a dyad is estimated to have an edge weight of 0.2, but the detectability of one of the individuals is only 0.5, then the estimate can be corrected upwards.

Another aspect that is not addressed in the current work is the issue of non-independence in the edge weights of different dyads. For example, it is possible that individual A associates with B only when B is associating with C. Because our Bayesian inference method updates edge weights independently of one another, it would break up such relationships. Direct bootstrapping does not suffer as much from this limitation in the context of estimating uncertainty because observations of the whole network are kept together. However, bootstrapped data may also retain spurious relationships that happened to occur within the observation period, especially when the number of observations is limited. A potentially powerful improvement to the Bayesian inference method could be to incorporate information about high-level patterns of interaction, such as incorporating information about clique membership and gregariousness. As our ability to collect large amounts of data on association patterns in animal societies improves (for example through the use of automated tracking technologies), these types of analyses might become feasible [17,18]. Incorporating information about higher-level processes (such as triads) could provide significantly more information about our observations and the processes that generate social structure. For example, if A, B and C are almost always observed together, then seeing A and B together without C might suggest that C was missed in the observation. Progress is being achieved in developing models that can quantify a range of variables representing higher-order processes [17,18]. For example, in animal social networks, one useful variable to estimate will be observability, as discussed above. The Bayesian approach has previously been shown to be useful for estimating the likelihood of missing edges in human data [16], but it is unlikely that the full potential of this method has been properly explored.

Finally, many social systems are only stable for short periods of time. Capturing the dynamics of social network structure could yield novel insight across a range of biological systems [2,35,36]. Bayesian networks could provide a tool to quantify the shifting patterns of association or interaction rates. Identifying periods of time when the 95% credible intervals for the edge weight of a given dyad no longer overlap could provide candidate time periods for when the relationship between individuals has changed. For example, if a dyad is observed 20 times, associating frequently during the first half of observations but never during the second, then at some point the intervals for observations beyond the 10th will no longer overlap the interval at the 10th. This way, a network built from Bayesian inference could identify candidate periods of transition without having to define an *a priori* set of bins or resolution for the dynamic network.

## 5. Conclusion

Our results suggest that Bayesian inference of edge weights is a useful tool both for measuring the uncertainty associated with edge weights within networks and for determining whether our estimates are likely to be reliable given how many times edges have been sampled. Importantly, we have also made some advances towards developing a tool to estimate how good a set of observations are at capturing real social structure. Technology increasingly facilitates capturing more precise data on animal associations. Studies that capture the complete set of dyadic associations will be useful for exploring how robust our measurements of network properties are under different levels of sampling [37]. Our results highlight the importance and utility of considering the uncertainty and stability of network measurements when drawing conclusions about social structure.

## Supplementary Material

1. Supplemental information and results

## Supplementary Material

2. Source code for evaluation of the method

## Supplementary Material

3. Source code for evaluating the method using a single simulated network

## Supplementary Material

4. Source code for evaluating the method using an empirical dataset.

## Data accessibility

All of the R codes for performing the analyses contained in this paper are available as the electronic supplementary material. The data from our empirical example are available as part of the *asnipe* library in R.

## References

[1] Farine DR, Garroway CJ, Sheldon BC 2012 Social network analysis of mixed-species flocks: exploring the structure and evolution of interspecific social behaviour. Anim. Behav. 84, 1271–1277. (doi:10.1016/J.Anbehav.2012.08.008)

[2] Pinter-Wollman N *et al* 2013 The dynamics of animal social networks: analytical, conceptual, and theoretical advances. Behav. Ecol. 25, 242–255. (doi:10.1093/beheco/art047)

[3] Sih A, Hanser SF, McHugh KA 2009 Social network theory: new insights and issues for behavioral ecologists. Behav. Ecol. Sociobiol. 63, 975–988. (doi:10.1007/S00265-009-0725-6)

[4] Wey T, Blumstein DT, Shen W, Jordan F 2008 Social network analysis of animal behaviour: a promising tool for the study of sociality. Anim. Behav. 75, 333–344. (doi:10.1016/J.Anbehav.2007.06.020)

[5] Farine DR, Whitehead H 2015 Constructing, conducting, and interpreting animal social network analysis. J. Anim. Ecol. 84, 1144–1163. (doi:10.1111/1365-2656.12418) 26172345 10.1111/1365-2656.12418 PMC4973823

[6] Formica VA, Wood CW, Larsen WB, Butterfield RE, Augat ME, Hougen HY, Brodie ED 2012 Fitness consequences of social network position in a wild population of forked fungus beetles (*Bolitotherus cornutus*). J. Evol. Biol. 25, 130–137. (doi:10.1111/j.1420-9101.2011.02411.x) 22092581 10.1111/j.1420-9101.2011.02411.x

[7] Wey TW, Burger JR, Ebensperger LA, Hayes LD 2013 Reproductive correlates of social network variation in plurally breeding degus (*Octodon degus*). Anim. Behav. 85, 1407–1414. (doi:10.1016/j.anbehav.2013.03.035) 24511149 10.1016/j.anbehav.2013.03.035 PMC3914217

[8] James R, Croft DP, Krause J 2009 Potential banana skins in animal social network analysis. Behav. Ecol. Sociobiol. 63, 989–997. (doi:10.1007/S00265-009-0742-5)

[9] Lusseau D, Whitehead H, Gero S 2008 Incorporating uncertainty into the study of animal social networks. Anim. Behav. 75, 1809–1815. (doi:10.1016/J.Anbehav.2007.10.029)

[10] Whitehead H 2008 Analyzing animal societies. Chicago, IL: University of Chicago Press.

[11] Snijders TAB 2011 Statistical models for social networks. Annu. Rev. Sociol. 37, 131–153. (doi:10.1146/annurev.soc.012809.102709)

[12] Croft DP, James R, Krause J 2008 Exploring animal social networks. Princeton, NJ: Princeton University Press.

[13] Farine DR 2015 Proximity as a proxy for interactions: issues of scale in social network analysis. Anim. Behav. 104, e1–e5. (doi:10.1016/j.anbehav.2014.11.019)

[14] Cairns SJ, Schwager SJ 1987 A comparison of association indexes. Anim. Behav. 35, 1454–1469. (doi:10.1016/S0003-3472(87)80018-0)

[15] Efron B, Tibshirani RJ 1994 An introduction to the bootstrap. Boca Raton, FL: Chapman and Hall/CRC.

[16] Butts CT 2003 Network inference, error, and informant (in)accuracy: a Bayesian approach. Soc. Networks 25, 103–140. (doi:10.1016/S0378-8733(02)00038-2)

[17] Everitt RG 2012 Bayesian parameter estimation for latent Markov random fields and social networks. J. Comp. Graph. Stat. 21, 940–960. (doi:10.1080/10618600.2012.687493)

[18] Hunter DR, Krivitsky PN, Schweinberger M 2012 Computational statistical methods for social network models. J. Comp. Graph. Stat. 21, 856–882. (doi:10.1080/10618600.2012.732921) 10.1080/10618600.2012.732921 PMC3697157 23828720

[19] Whitehead H 2009 SOCPROG programs: analysing animal social structures. Behav. Ecol. Sociobiol. 63, 765–778. (doi:10.1007/S00265-008-0697-Y)

[20] Farine DR 2013 Animal social network inference and permutations for ecologists in R using *asnipe*. Methods Ecol. Evol. 4, 1187–1194. (doi:10.1111/2041-210X.12121)

[21] R Development Core Team. 2013 R: a language and environment for statistical computing. Vienna, Austria: R Foundation for Statistical Computing.

[22] Murphy KP 2002 Machine learning: a probabilistic perspective. Cambrige, MA: MIT Press.

[23] Agresti A 2002 Categorical data analysis, 2nd edn Hoboken, NJ: John Wiley and Sons, Inc.

[24] Psorakis I, Roberts SJ, Rezek I, Sheldon BC 2012 Inferring social network structure in ecological systems from spatio-temporal data streams. J. R. Soc. Interface 9, 3055–3066. (doi:10.1098/rsif.2012.0223) 22696481 10.1098/rsif.2012.0223 PMC3479900

[25] Psorakis I 2015 Inferring social structure from temporal data. Behav. Ecol. Sociobiol. 69, 857–866. (doi:10.1007/s00265-015-1906-0)

[26] Aplin LM, Farine DR, Morand-Ferron J, Cockburn A, Thornton A, Sheldon BC 2015 Experimentally induced innovations lead to persistent culture via conformity in wild birds. Nature 518, 538–541. (doi:10.1038/nature13998) 25470065 10.1038/nature13998 PMC4344839

[27] Aplin LM, Farine DR, Morand-Ferron J, Cole EF, Cockburn A, Sheldon BC 2013 Individual personalities predict social behaviour in wild networks of great tits (*Parus major*). Ecol. Lett. 16, 1365–1372. (doi:10.1111/ele.12181) 24047530 10.1111/ele.12181

[28] Farine DR, Sheldon BC 2015 Selection for territory acquisition is modulated by social network structure in a wild songbird. J. Evol. Biol. 28, 547–556. (doi:10.1111/jeb.12587) 25611344 10.1111/jeb.12587 PMC4406129

[29] Franks DW, Ruxton GD, James R 2010 Sampling animal association networks with the gambit of the group. Behav. Ecol. Sociobiol. 64, 493–503. (doi:10.1007/s00265-009-0865-8)

[30] Farine DR *et al* 2015 The role of social and ecological processes in structuring animal populations: a case study from automated tracking of wild birds. R. Soc. open sci. 2, 150057 (doi:10.1098/rsos.150057) 26064644 10.1098/rsos.150057 PMC4448873

[31] Bejder L, Fletcher D, Brager S 1998 A method for testing association patterns of social animals. Anim. Behav. 56, 719–725. (doi:10.1006/anbe.1998.0802) 9784222 10.1006/anbe.1998.0802

[32] Croft DP, Madden JR, Franks DW, James R 2011 Hypothesis testing in animal social networks. Trends Ecol. Evol. 26, 502–507. (doi:10.1016/J.Tree.2011.05.012) 21715042 10.1016/j.tree.2011.05.012

[33] Farine DR 2014 Measuring phenotypic assortment in animal social networks: weighted associations are more robust than binary edges. Anim. Behav. 89, 141–153. (doi:10.1016/j.anbehav.2014.01.001)

[34] Whitehead H, Bejder L, Ottensmeyer CA 2005 Testing association patterns: issues arising and extensions. Anim. Behav. 69, e1–e6. (doi:10.1016/j.anbehav.2004.11.004)

[35] Blonder B, Wey TW, Dornhaus A, James R, Sih A 2012 Temporal dynamics and network analysis. Methods Ecol. Evol. 3, 958–972. (doi:10.1111/J.2041-210x.2012.00236.X)

[36] Hobson EA, Avery ML, Wright TF 2013 An analytical framework for quantifying and testing patterns of temporal dynamics in social networks. Anim. Behav. 85, 83–96. (doi:10.1016/J.Anbehav.2012.10.010)

[37] Haddadi H, King AJ, Wills AP, Fay D, Lowe J, Morton AJ, Hailes S, Wilson AM 2011 Determining association networks in social animals: choosing spatial–temporal criteria and sampling rates. Behav. Ecol. Sociobiol. 65, 1659–1668. (doi:10.1007/S00265-011-1193-3)

