# Supplementary material for: Estimating uncertainty and reliability of social network data using Bayesian inference
Source: R Soc Open Sci. 2015 Sep 16;2(9):150367. doi: 10.1098/rsos.150367 (PMC4593693; doi:10.1098/rsos.150367)
Supplement: 1. Supplemental information and results [file rsos150367supp1.pdf]

***Farine & Strandburg-Pershkin*: Estimating uncertainty and reliability of network data using Bayesian inference**

Obtaining priors using maximum likelihood estimation

To begin, for a single edge weight, assume we have observed d co-occurrences out of s potential observations. We can then express the likelihood of the data given a prior distribution described by a parameter set $\{a, b\}$ as

$$P(d|s,a,b) = \int_0^1 P(d|s,x,a,b)P(x|s,a,b)dx$$

where here we are integrating over the unknown edge weight, $x \in [0,1]$. For iid binary observations and a beta-distributed prior, $\beta(x; a, b)$, we have

$$P(d|s,a,b) = \binom{s}{d} \int_0^1 x^d (1-x)^{s-d} \beta(x; a, b) dx$$

$$= \binom{s}{d} \int_0^1 x^d (1-x)^{s-d} \frac{x^{a-1}(1-x)^{b-1}}{Z(a,b)}$$

where *Z(a,b)* is the beta function (the normalization constant to the beta distribution) defined as

$$Z(a,b) = \int_0^1 y^{a-1}(1-y)^{b-1} dy$$

Simplifying yields

$$P(d|s,a,b) = \binom{s}{d} \frac{Z(a+d, b+s-d)}{Z(a,b)}$$

for a single edge weight. Across all n edge weights (assumed iid), $x_1, x_2, \dots x_n$, we then have

$$P(d_1, d_2 \dots d_n | s_1, s_2, \dots s_n, a, b) = \int_0^1 P(d_1, d_2 \dots d_n | s_1, s_2, \dots s_n, x_1, x_2, \dots x_n, a, b) P(x|a,b) dx$$

$$= \prod_{i=1}^{n} \binom{s_i}{d_i} \frac{Z(a+d_i, b+s_i-d_i)}{Z(a,b)}$$

By maximizing this product with respect to $\{a, b\}$, we obtain the maximum likelihood parameters (a^*, b^*). The prior distribution for each edge weight is then defined by $\beta(x; a^*, b^*)$.

Supplemental Figures

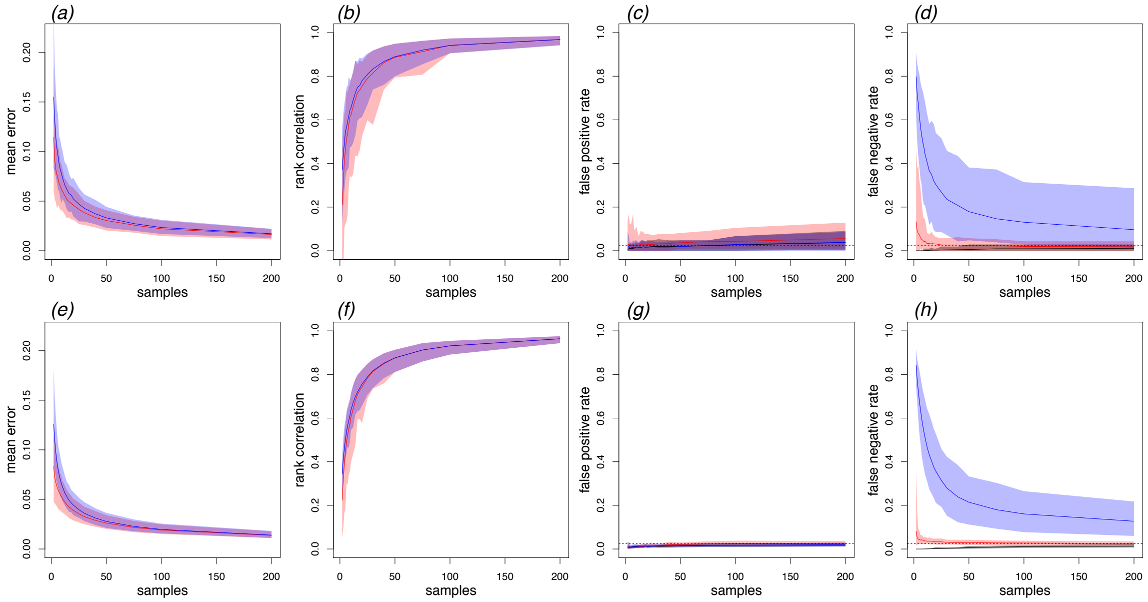

Figure S1: Summary of edge weight accuracy and reliability of uncertainty estimates for small (N=15, top row) and large (N=50, bottom row) networks with no cliques using Bayesian (red) and b-SRI (blue) methods from 100 simulated networks (shown are the median and 95% range). Mean error (a,e) is the mean absolute difference between the estimated edge weight and the input edge weight in the 'real' network for increasing number of samples (x-axis). Rank correlation (b,f) is the correlation in the order of edge weight values, which we call relative accuracy. False positive rate (c,g) is the proportion of edges in the 'real' network that fall below the 95% confidence (bootstrap in blue and Clopper-Pearson in grey) and credible (Bayesian in red) intervals. False negative rate (d,h) is the proportion of edges in the 'real' network that fall above the 95% range for each method for estimating uncertainty.

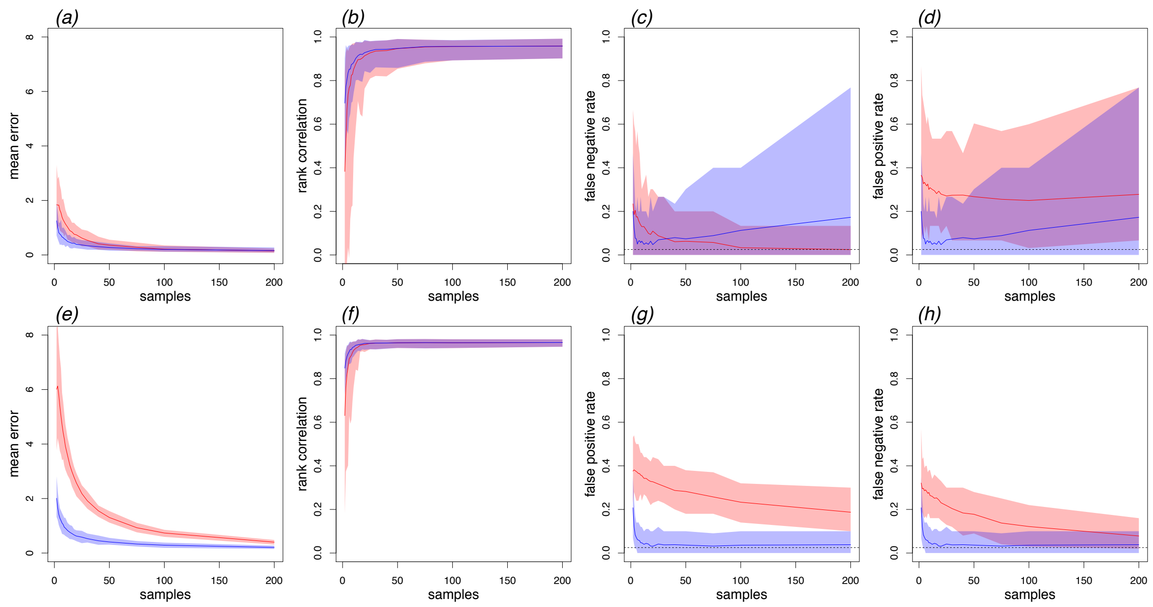

Figure S2: Summary of degree accuracy and reliability of uncertainty estimates for small (N=15, top row) and large (N=50, bottom row) networks with no cliques using Bayesian (red) and b-SRI (blue) methods from 100 simulated networks (shown are the median and 95% range). Mean error (a,e) is the mean absolute difference between the estimated node degree and the degree in the 'real' network for increasing number of samples (x-axis). Rank correlation (b,f) is the correlation in the order of degree scores, which we call relative accuracy. False positive rate (c,g) is the proportion of node degree values in the 'real' network that fall below the 95% confidence (bootstrap in blue and Clopper-Pearson in grey) and credible (Bayesian in red) intervals. False negative rate (d,h) is the proportion of node degree values in the 'real' network that fall above the 95% range for each method for estimating uncertainty.

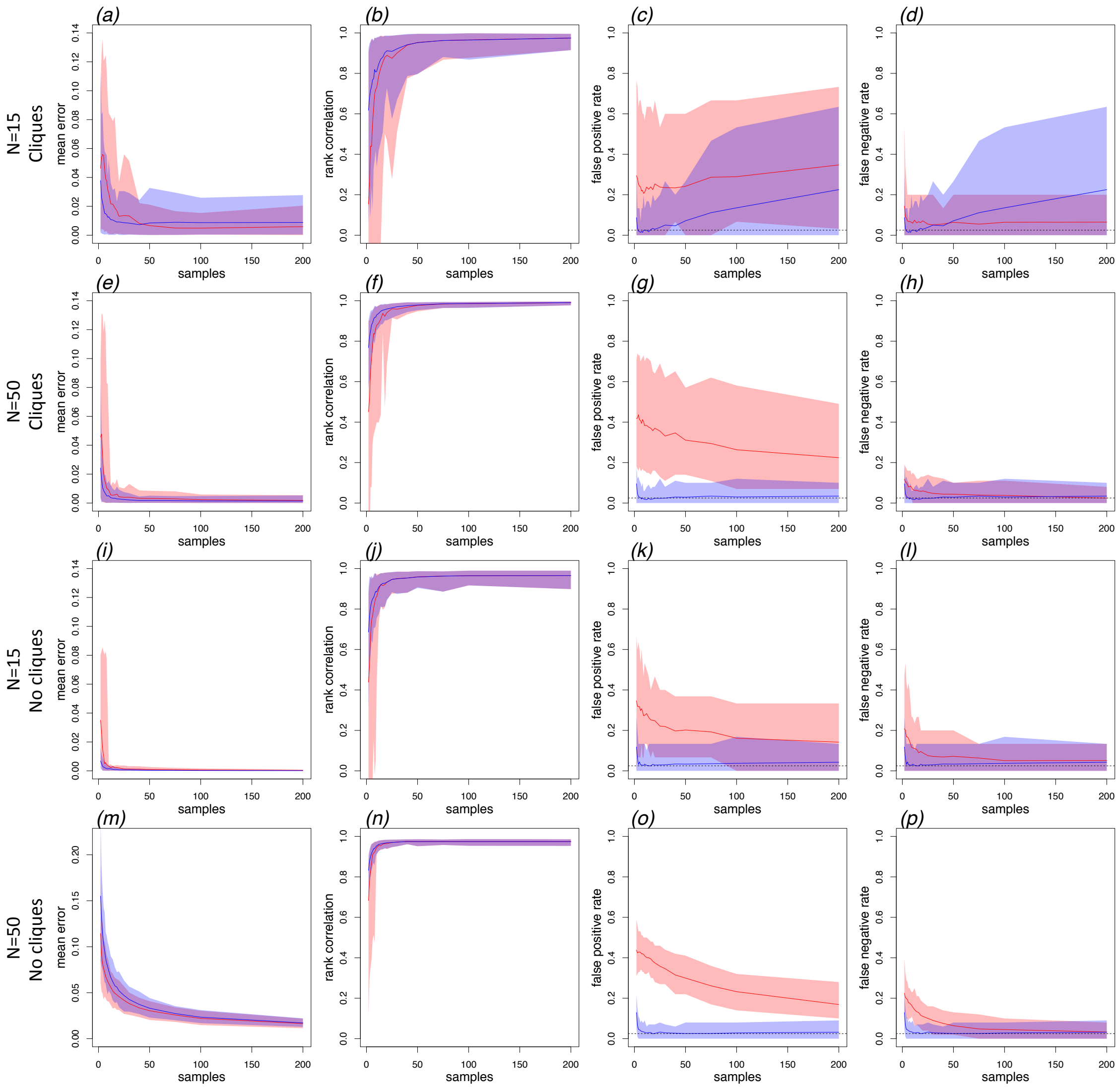

Figure S3: Summary of eigenvector centrality accuracy and reliability of uncertainty estimates for small (N=15, 1st and 3rd rows) and large (N=50, 2nd and 4th rows) networks with cliques (top two rows) and no cliques (bottom two rows) using Bayesian (red) and b-SRI (blue) methods from 100 simulated networks (shown are the median and 95% range). Mean error (a,e) is the mean absolute difference between the estimated node eigenvector centrality and the eigenvector centrality in the 'real' network for increasing number of samples (x-axis). Rank correlation (b,f) is the correlation in the order of eigenvector centrality scores, which we call relative accuracy. False positive rate (c,g) is the proportion of node eigenvector centrality values in the 'real' network that fall below the 95% confidence (bootstrap in blue and Clopper-Pearson in grey) and credible (Bayesian in red) intervals. False negative rate (d,h) is the proportion of node eigenvector centrality values in the 'real' network that fall above the 95% range for each method for estimating uncertainty.

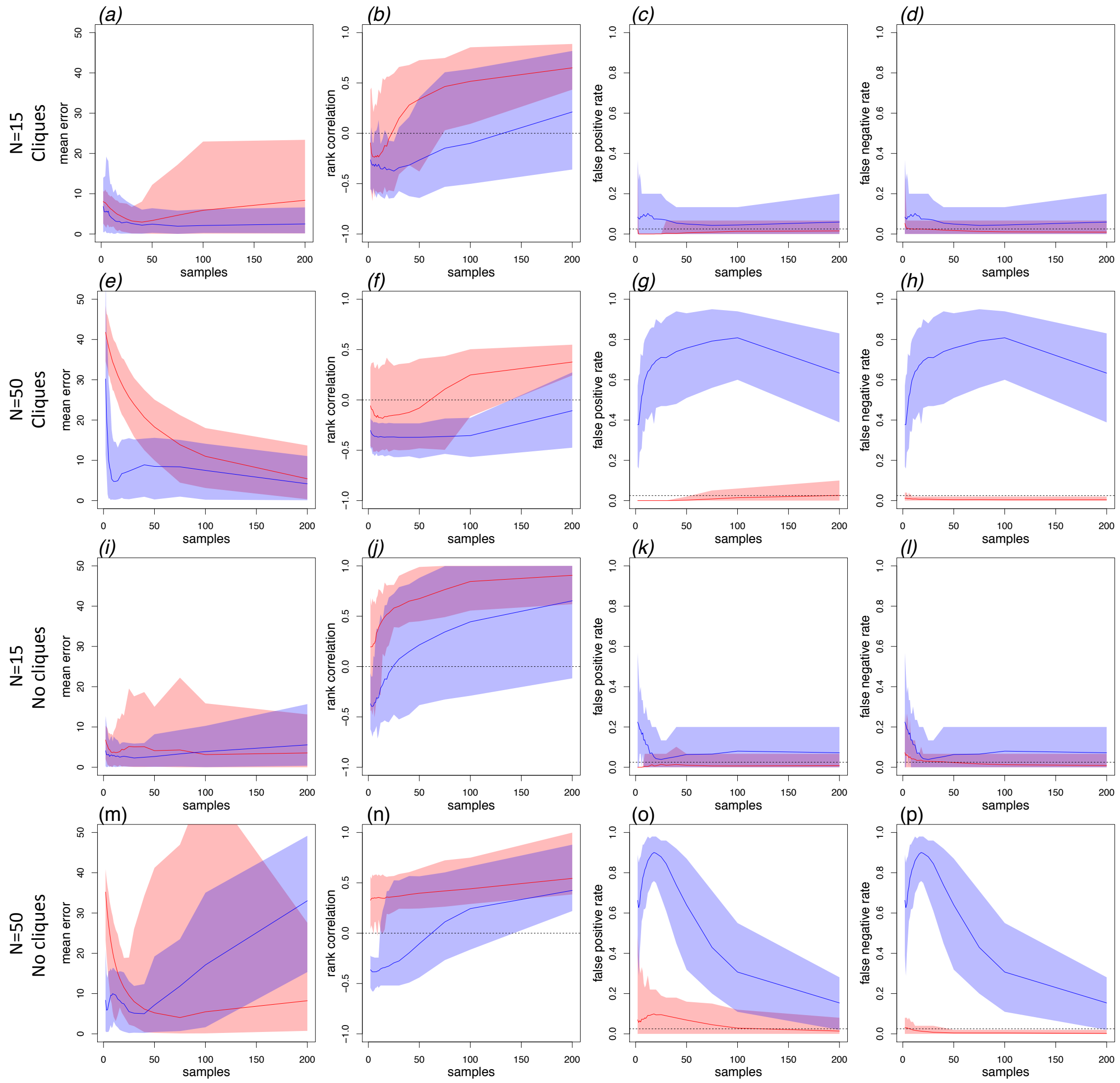

Figure S4: Summary of betweenness accuracy and reliability of uncertainty estimates for small (N=15, 1st and 3rd rows) and large (N=50, 2nd and 4th rows) networks with cliques (top two rows) and no cliques (bottom two rows) using Bayesian (red) and b-SRI (blue) methods from 100 simulated networks (shown are the median and 95% range). Mean error (a,e) is the mean absolute difference between the estimated node betweenness and the betweenness in the 'real' network for increasing number of samples (x-axis). Rank correlation (b,f) is the correlation in the order of betweenness scores, which we call relative accuracy. False positive rate (c,g) is the proportion of node betweenness values in the 'real' network that fall below the 95% confidence (bootstrap in blue and Clopper-Pearson in grey) and credible (Bayesian in red) intervals. False negative rate (d,h) is the proportion of node betweenness values in the 'real' network that fall above the 95% range for each method for estimating uncertainty.

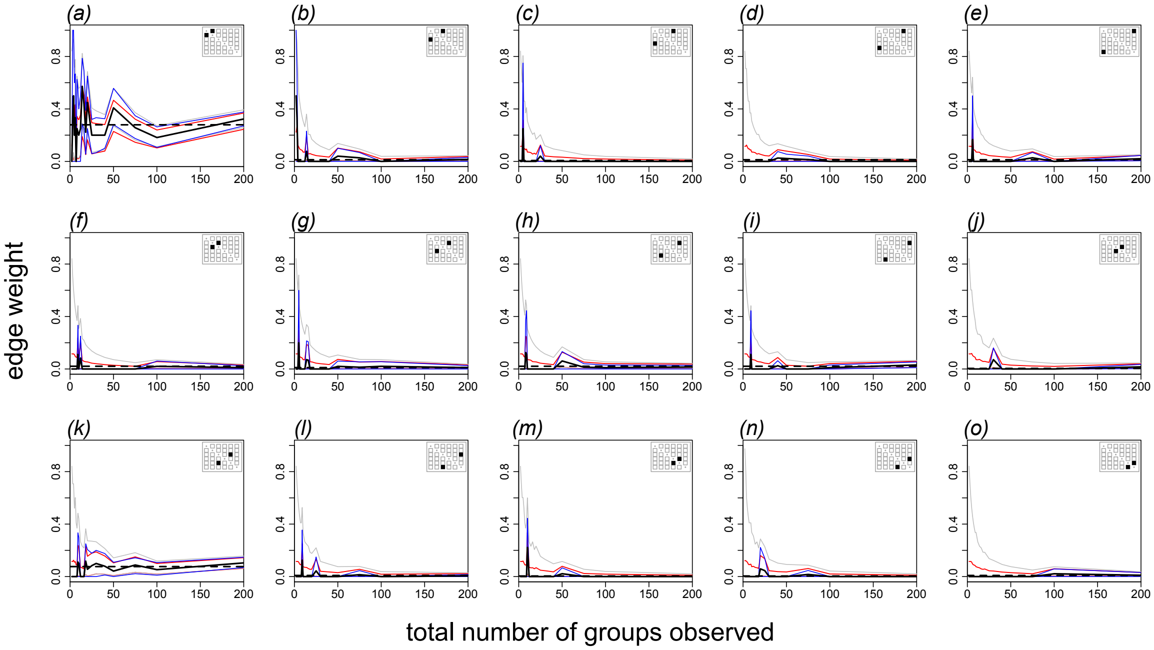

Figure S5: Example of estimated uncertainty ranges for 15 edges from one simulated network (N=15) with cliques (using the same algorithm as in Figure 4) based on the Bayesian method (red), the bootstrap method (blue) and the Clopper-Pearson method (grey). Each plot shows the 95% confidence or credible intervals and the estimated edge weight based on the simple ratio index (black line), for increasing number of samples (up to 200). The matrix in the top-right of each plot identifies which edge in the top-left 5x5 corner of the association matrix (in this case, a symmetrical matrix). Dashed black lines in each plot indicate the true value of the edge weight.

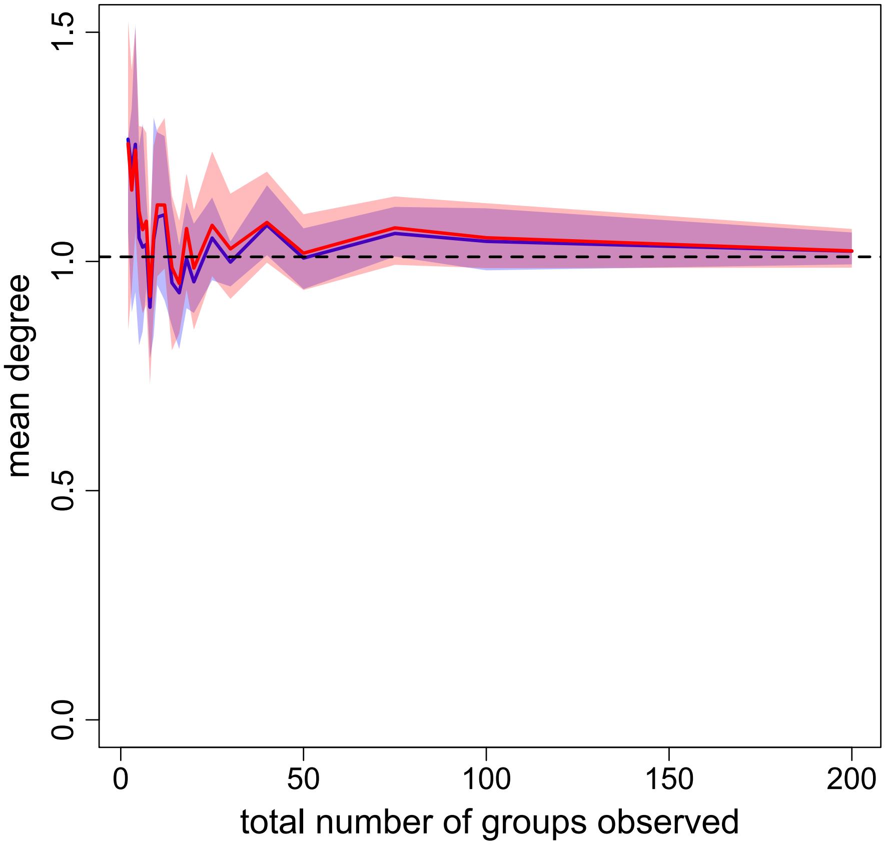

Figure S6: Estimated mean degree (solid lines) and uncertainty ranges (shaded regions) using either the Bayesian (red) or b-SRI (blue) method in a simulated network comprising 15 individuals and cliques, for increasing number of samples. The mean degree first decreases and then stabilizes as more observations are added. This suggests that the network was adequately sampled after approximately 30 samples. Note that the difference between this network and the empirical network (Figure 5, where the mean degree increases as more samples are added) is because all nodes are observed in each sample of the simulated network.
